# NF-Y Overexpression in Liver Hepatocellular Carcinoma (HCC)

**DOI:** 10.3390/ijms21239157

**Published:** 2020-12-01

**Authors:** Eugenia Bezzecchi, Mirko Ronzio, Roberto Mantovani, Diletta Dolfini

**Affiliations:** Dipartimento di Bioscienze, Università degli Studi di Milano, Via Celoria 26, 20133 Milano, Italy; eugenia.bezzecchi@unimi.it (E.B.); mirko.ronzio@unimi.it (M.R.); mantor@unimi.it (R.M.)

**Keywords:** HCC, NF-Y, Transcription Factors, TCGA, CCAAT box

## Abstract

NF-Y is a pioneer trimeric transcription factor formed by the Histone Fold Domain (HFD) NF-YB/NF-YC subunits and NF-YA. Three subunits are required for DNA binding. CCAAT-specificity resides in NF-YA and transactivation resides in Q-rich domains of NF-YA and NF-YC. They are involved in alternative splicing (AS). We recently showed that NF-YA is overexpressed in breast and lung carcinomas. We report here on the overexpression of all subunits in the liver hepatocellular carcinoma (HCC) TCGA database, specifically the short NF-YAs and NF-YC2 (37 kDa) isoforms. This is observed at all tumor stages, in viral-infected samples and independently from the inflammatory status. Up-regulation of NF-YAs and NF-YC, but not NF-YB, is associated to tumors with mutant p53. We used a deep-learning-based method (DeepCC) to extend the partitioning of the three molecular clusters to all HCC TCGA tumors. In iCluster3, CCAAT is a primary matrix found in promoters of up-regulated genes, and cell-cycle pathways are enriched. Finally, clinical data indicate that, globally, only NF-YAs, but not HFD subunits, correlate with the worst prognosis; in iCluster1 patients, however, all subunits correlate. The data show a difference with other epithelial cancers, in that global overexpression of the three subunits is reported and clinically relevant in a subset of patients; yet, they further reinstate the regulatory role of the sequence-specific subunit.

## 1. Introduction

Liver cancer is one of the most widespread types of cancer worldwide. Its major form is Hepatocellular Carcinoma (HCC) [1,2]. There are several risk factors in developing HCC, such as chronic viral infections (HBV and HCV), alcohol abuse, autoimmune hepatitis and metabolic diseases [3,4]. These conditions are believed to cause chronic inflammation, which entails continuous necrosis and tissue regeneration. In turn, this process causes the fixation of cumulative genetic and epigenetic changes, finally resulting in cancer [5]. In addition to the identification of mutated genes, alterations of DEG (Differentially Expressed Genes) have been determined in many studies: RNA-seq analysis allowed TCGA to classify HCC in three subtypes, iCluster1/2/3 [6]; these clusters correspond well to the classes previously identified based on analysis of large databases of microarray profiling [7].

In general, cellular transformation causes stable changes in the patterns of gene expression, the focal point of which is the regulation of transcriptional initiation, as dictated by sequence-specific Transcription Factors (TFs) [8]. Hence, the promoters of genes overexpressed in cancers have been scanned for TFBSs (Transcription Factor Binding Sites), and the CCAAT box was identified as an enriched matrix in profiling analysis of >60.000 tumor samples [9].

The CCAAT element is relatively widespread in human promoters and is therefore functionally important [10]. The CCAAT-binding TF is NF-Y, a heterotrimer formed by the Histone Fold Domain (HFD) dimer NF-YB/NF-YC and by NF-YA [11]. Recently, CCAAT was included among elements that govern the selection of TSS (Transcriptional Start Sites) [12]. NF-Y has large Trans-Activation Domains (TADs) located at the N-terminal of NF-YA and at the C-terminal of NF-YC [13,14,15,16]. Both TADs are rich in Gln and hydrophobic amino acids and involved in Alternative Splicing events. NF-YA comes in two major isoforms, NF-YAl, comprising exon-3 sequences, and NF-YAs, lacking them. NF-YC has isoforms of 37, 48 and 50 kD. The evolutionarily conserved parts of each subunit, imparting heterotrimerization and DNA-binding, are present in all isoforms. As for the NF-Y regulome, at least two pathways are under NF-Y control: cell-cycle and metabolic genes [17,18,19] reviewed in [20].

Despite the original categorization as “ubiquitous” TF, NF-Y subunits are regulated in different tissues, including in liver: notably NF-YB by cell-size [21] and NF-YA by proliferation [22] and c-AMP treatment [23]. Data from conditional liver-specific KO mice reported severe organ-specific, as well as more general, alterations [23,24]. As for targeted genes, initial studies on the Albumin promoter [25,26,27] were followed by a long list of hepatic genes requiring NF-Y/CCAAT for function [28], among which SREBPs, FAS, HKII and SOX9 are involved in development of hepatocellular carcinomas [29,30,31,32]. NF-Y was shown to promote activation of the HBV Protein S and Cp promoters [33,34]; finally, the oncogenic HBV X transactivator activates the AKR1C1 gene through an NF-Y site [35].

In general, data on the mRNA levels of NF-Y subunits in cancer are only recently emerging. In initial reports on individual cohorts of ovarian, breast and gastric cancer, it was shown that NF-YA is overexpressed [36,37,38,39,40]. This led to a systematic investigation of RNA-seq experiments present in TCGA and in individual GEO datasets. We have completed the analysis of Breast Carcinomas (BRCA), Lung Squamous Cells Carcinomas (LUSC) and Adenocarcinomas (LUAD): NF-YA, but not HFD subunits, is overexpressed, with relevant clinical consequences [41,42,43]. We report here a different pattern in liver hepatocarcinomas.

## 2. Results

### 2.1. NF-Y Subunits Are Overexpressed in HCC

**Figure 1:**
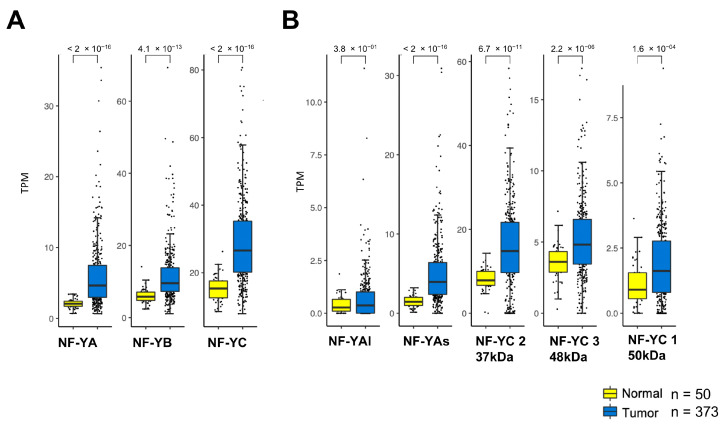
NF-Y subunits are overexpressed in HCC (Hepatocellular Carcinoma). (**A**) Box plots of expression levels of the three NF-Y subunits at gene level in the TCGA-HCC dataset, measured in TPMs (Transcript per Millions). (**B**) In the same way as A, the NF-YA—NF-YAs, NF-YAl—and NF-YC isoforms levels were analyzed.

NF-YA is elevated in numerous epithelial tumors [41]. We downloaded the datasets of 373 liver hepatocarcinoma RNA-seq and 50 normal counterparts of TCGA [6]: analysis of expression levels of the NF-Y subunits shows that all are overexpressed, with very significant *p* values (10^−13/16^) (Figure 1A). We then analyzed the different isoforms of NF-YA and NF-YC: NF-YAs, the predominant isoform, substantially increased (*p* value 10^−16^) (Figure 1B). The expression of the major isoform of NF-YC—37 kD—is also primarily increased in tumors (*p* value 10^−11^), more than the less expressed 48 and 50 kD isoforms (*p* value 10^−04/06^) (Figure 1B).

### 2.2. Expression of NF-Y Isoforms According to Viral Infection, Stage and Inflammation of HCC

**Figure 2:**
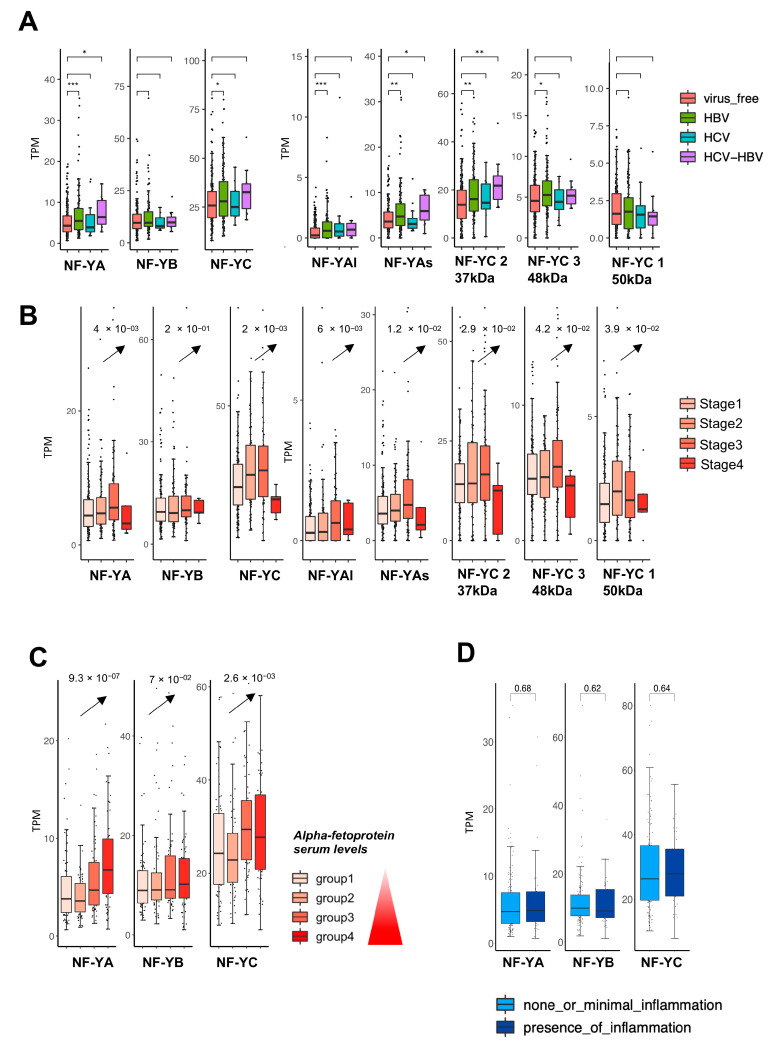
NF-Y expression levels according to viral infection, pathological stage and inflammation status. (**A**) Box plots of expression levels of NF-Y subunits at gene and isoforms levels in virus-positive and virus-free TCGA samples (stars indicate *p* value significance according to the following criteria: *** < 0.001; ** < 0.01; * < 0.05). (**B**) Box plots of expression levels of the three NF-Y subunits at gene and isoform levels divided according to pathological stage. (**C**) Box plot of NF-Y subunits expression levels after stratification of samples according to alfa-fetoprotein serum levels. (**D**) Box plots of NFY subunits in presence of inflammation compared with low/minimal inflammation.

HCC samples are classified according to (i) infections by the HBV and/or HCV viruses (Table S1), (ii) stage, and (iii) presence of intratumoral inflammation (Table S2). First, we analyzed the subunit levels in the RNA-seq of HBV- and/or HCV-positive tumors: moderate—*p* value <10^−2/−3^—statistically increased expression of NF-YA and NF-YC is detected in HBV^+^ and HBV^+^/HCV^+^ but not in HCV^+^ tumors (Figure 2A, left panels); NF-YAs and NF-YC 37 kD are mainly responsible for this increase (Figure 2A, right panels). All subunits increased in the four Stages (I–IV), and notably this already occurred at Stage I, with an ascending trend toward Stage IV (Figure 2B, left panels); at isoform level, again, we found a clear trend in NF-YAs and NF-YC 37 kD (Figure 2B, right panels). We also stratified samples based on the serum levels of one of the most widely used serum biomarkers for HCC: Alpha-fetoprotein (AFP). NF-YA and NF-YC showed a clear increasing expression trend that followed the increase of AFP serum levels (Figure 2C). NF-YAs, being the most abundant isoform, showed the same significant increasing trend as the NF-YA total (Figure S1). As for inflammation, tumors were classified as none/minimal or present: the NF-Y subunits did not show significant overexpression in the presence of inflammatory conditions (Figure 2D). As a control, we checked the 18 genes of the HCC Tumor Inflammatory Signature (TIS) as determined in a previous study [44]. As expected, all robustly increased in the inflammatory cohort (Figure S2A). We divided the samples in two groups: none or minimal and presence of inflammation. We stratified the samples according to NF-Y subunits expression and we observed the expression trend of TIS genes. As for the none or minimal inflammation group, only STAT1 and CD276 show an increasing expression trend according to NF-Y subunits expression (Figure S2B). The same trend for STAT1 is also observed for the group with presence of inflammation (Figure S2C). Note that STAT1 is a direct target of NF-Y, and this could justify the correlation between the expression of NF-Y and STAT1. These data suggest that HBV infection has moderate impact on subunits levels, which are elevated at early stages and even without inflammation.

### 2.3. Partitioning of All TCGA HCC in iClusters According to DeepCC

**Figure 3:**
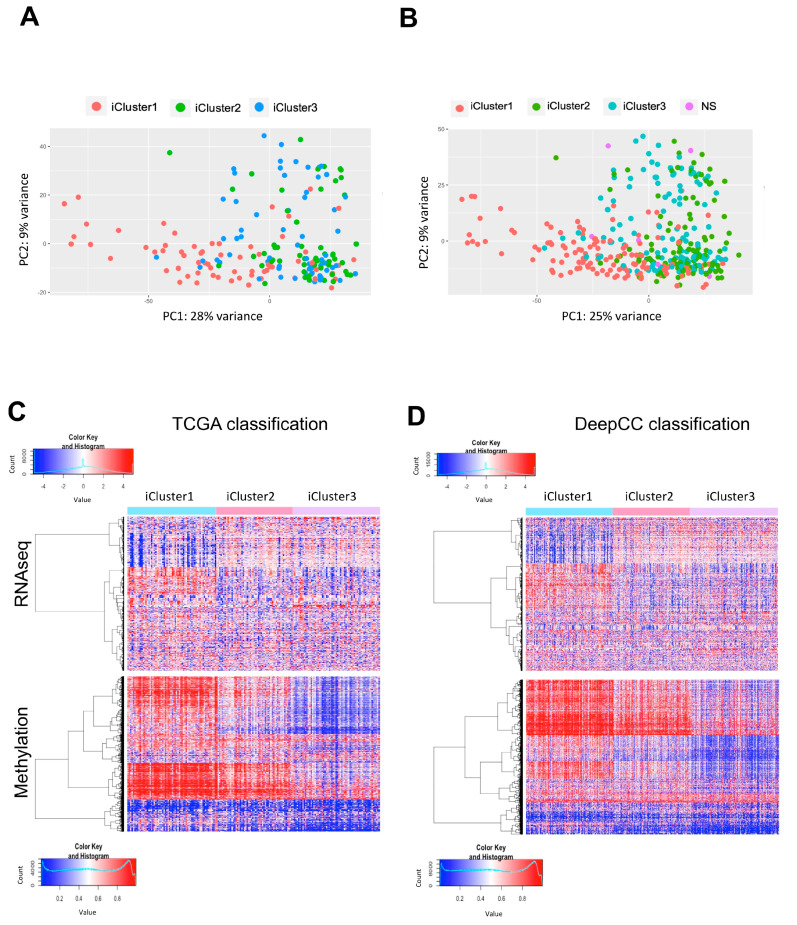
Partitioning of all TCGA HCC tumors in iClusters. (**A**) Principal Component Analysis of 183 tumor samples previously categorized by TCGA. (**B**) Principal Component Analysis (PCA) of all the 373 tumor samples partitioned in subtypes through DeepCC. (**C**) Gene mRNA expression (upper panel) and methylation profile (lower panel) in iClusters according to TCGA classification. (**D**) RNA-seq and methylation profile of DeepCC-derived iClusters. In both heatmaps, genes/methylation probes are rows and columns are tumor samples.

TCGA classified a portion (183 samples) of HCC in three iClusters, according to a panel of different features, including mutations, clinical data, vascularization, RNA-seq profilings, and methylation status [6]. Before proceeding with further analysis, we extended the iCluster classification to all tumors present in the HCC dataset. To do so, we took advantage of DeepCC, a deep-learning method for classification of tumors in subtypes [45]. Based on the previous classifications in iCluster1, iCluster2 and iCluster3, we fed the algorithm with the whole set of HCC RNA-seq data, avoiding genes whose standard deviation was equal to zero. Partitioning of 183/190 not-yet-classified HCC tumors was obtained (Table S2). The data shown in Figure 3 illustrate the robustness of our classification in comparison with the previous, partial one. First, Principal Component Analysis (PCA) shows that our global classification is no more variant, and indeed less so, than the previous one (Figure 3A,B, 25% vs. 28%). Stages and age-of-onset data show distributions similar to the previous classification (Figure S3A,B). Furthermore, mutations in the TP53 gene are also a feature found mainly in iCluster3, as shown in previous analysis (Figure S3C). Most importantly, the three iClusters are visible when considering data from both RNA-seq (Figure 3C,D, upper panels) and DNA methylomes (Figure 3C,D, lower panels). As shown in the heatmaps, iCluster-specific RNA-seq expression patterns are maintained in the complete classification (Figure 3C,D). The methylation patterns that distinguish iClusters are also verified in the 8000 random probes represented in Figure 3C,D. In summary, we are confident that our new, complete classification of all TCGA data in the iClusters is appropriate for further analysis.

### 2.4. Expression of NF-Y Subunits in iClusters

Overexpression of NF-Y subunits and/or isoforms could be limited to one or more of the clusters. We thus analyzed them separately. Figure S4A shows that the increase in expression of all subunits is visible in all clusters, but it is particularly robust for NF-YA and NF-YC in iCluster1 and for all subunits in iCluster3 (*p* values < 10^−15/16^). NF-YAs, but not NF-YAl, increased, especially in iCluster3; as for NF-YC, the 37kD and the less expressed 48 kD isoforms mostly increased, chiefly in iCluster3 (Figure S4B). We conclude that although the three subunits are overexpressed in all subtypes, iCluster1 and iCluster3 show the most robust overexpression of the NF-YAs and NF-YC 37 kDa subunits.

### 2.5. iCluster3 Differentially Expressed Genes (DEG) Have CCAAT in Promoters

**Figure 4:**
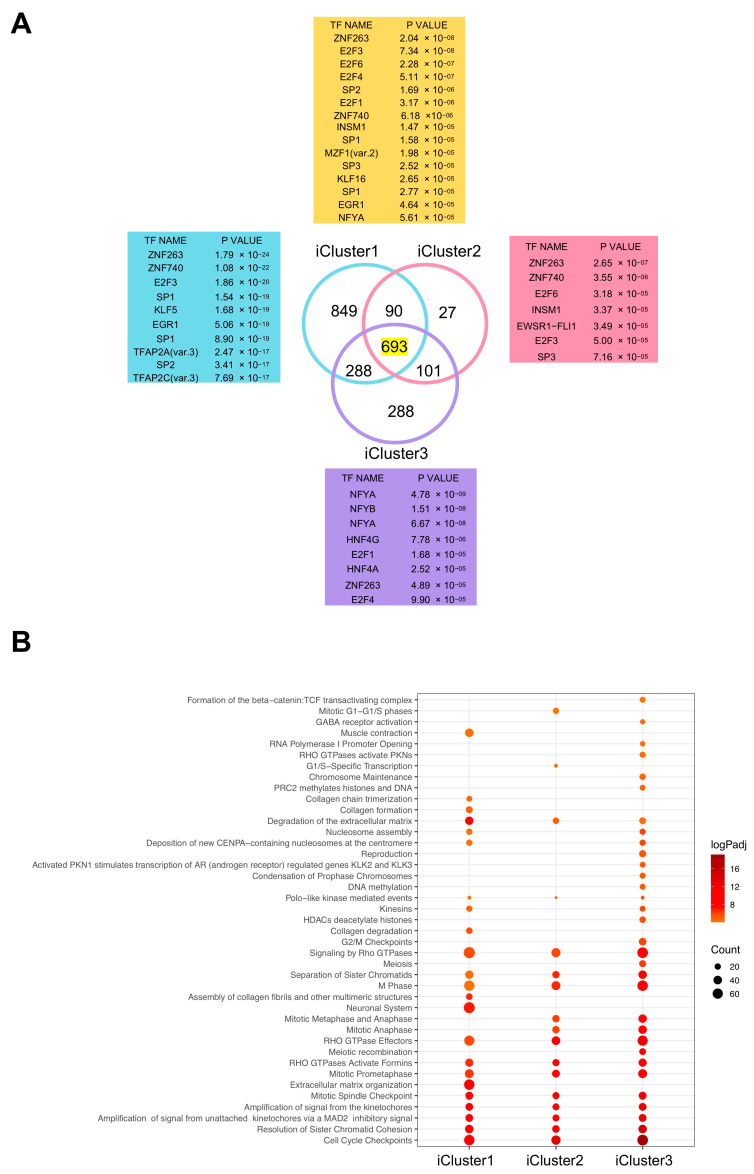
Analysis of HCC iCluster-specific gene expression. (**A**) Venn diagrams of Up-regulated genes in the three different iClusters and Transcription Factor Binding Sites (TFBS) enriched in their promoters, both in the core set and individually. (**B**) Dotplots of Reactome pathways enriched in upregulated gens of every iCluster.

We then compared the RNA-seq gene expression data of the three iClusters with those of normal samples. Using Log2 FC > 2, FDR < 0.01 thresholds, 1920, 911 and 1370 genes were overexpressed, and 463, 324 and 595 genes were down-regulated in iClusters1, iCluster2 and iCluster3, respectively. The lists of DEG in the three Clusters are presented in Table S3. A Venn diagram of common and cluster-specific up-regulated DEG is shown in Figure 4A. We analyzed their promoters (from −450 to +50 from the TSS) with Pscan software [46] to search for enriched TFBS. In iCluster1 and iCluster2, we recovered matrices of Zn Finger TFs (ZNF263, ZNF740, KLF5, Sp1) and E2Fs (E2F3, E2F6, E2F1); the NF-Y matrix (NFYA/NFYB) was absent (Figure 4A). In iCluster3, instead, the CCAAT matrix was at the top of the list of enriched TFBS, with significant *p* values. As for down-regulated genes, different matrices were found in the three iCusters, all unrelated to CCAAT (Figure S5). We also analyzed up-regulated DEG common to all three Clusters and found NF-YA among enriched TFBS (Figure 4A, upper panel). Next, we identified Gene Ontology terms in DEG using the KOBAS software; comparison among the terms enriched in the individual iClusters showed commonalities as well as specificities: *cell-cycle*, *G2/M*, *mitotic* and *M phase* terms were in all iClusters, particularly in iCluster3 (Figure 4B). iCluster1 was also enriched in *extracellular matrix* terms. We conclude that CCAAT-containing, up-regulated DEG are specifically enriched in the HCC iCluster3 cohort.

### 2.6. Analysis of NF-Y Subunit Levels in p53-Mutated HCC

**Figure 5:**
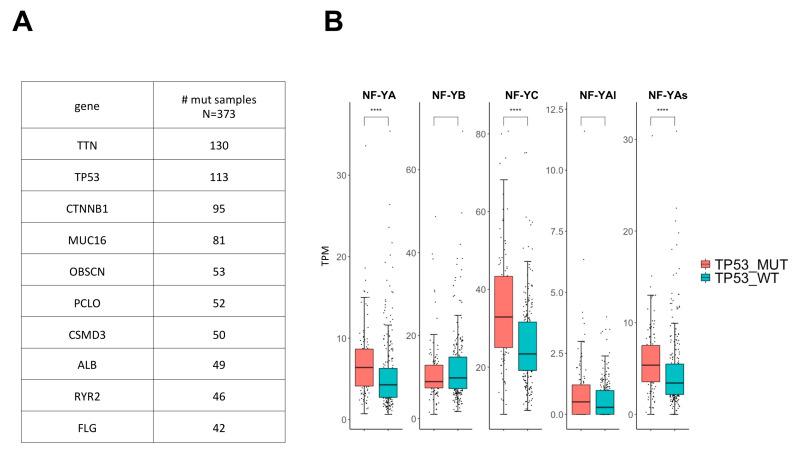
NF-Y subunits levels in HCC TP53^MUT^. (**A**) Table with the top ten HCC mutations. (**B**) Box plot of NF-Y subunits and NF-YA isoforms mRNA levels in TP53^MUT^ compared with TP53^WT^ samples (*p* value: **** < 0.0001).

Somatic mutations were analyzed in 373 HCC tumors by TCGA [6]. Most mutations were found in a relatively small number of samples: Figure 5A shows mutations present in more than 40 HCC samples. TP53 mutations were among the most abundant and were further considered; note, however, that the majority of HCC tumors were in the TP53 wt configuration (260/373). Another reason to focus on TP53-mutated tumors is that they are the hallmarks of iCluster3, enriched in CCAAT-box genes: indeed, 49% of HCC mutated in TP53 belonged to this iCluster (Figure S3C). When expression of the subunits was evaluated according to the TP53 status, a clear increase of NF-YA, notably NF-YAs and of NF-YC but not NF-YB, was indeed visible (Figure 5B).

### 2.7. Clinical Outcomes of NF-Y Overexpression in HCC

**Figure 6:**
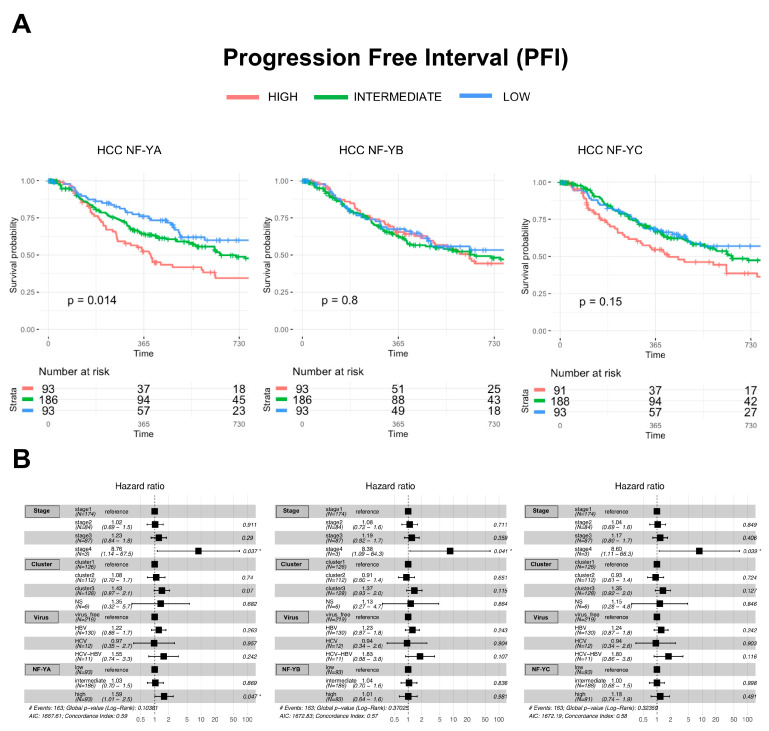
Clinical outcome of HCC tumors with different NF-Y subunits levels. (**A**) Progression-Free-Interval curves of HCC tumors with stratification according to quartiles distribution of NF-Y subunits expression (Intermediate, High and Low). (**B**) Hazard ratios of the sample cohorts partitioned according to Stage (I–IV), iClusters, viral infection and NF-Y levels (low is set as reference).

To assess the clinical relevance of NF-Y subunit overexpression, we first stratified all HCC samples according to quartiles distribution: High, Intermediate and Low mRNA levels of NF-YA, NF-YB and NF-YC; thereafter, we considered the outcome of patients. Performing Progression Free Interval (PFI) analysis, NF-YA^high^ tumors, but not NF-YB^high^ or NF-YC^high^, are statistically associated with a poor outcome of the disease (Figure 6A). This was confirmed by Hazard Ratio analysis, which included other parameters of HCC (Figure 6B). Analyzing the data according to iCluster categorizations, we noticed a worsening of the prognosis only in iCluster1 patients (Figure S6A), but not iClusters 2 or 3 (Figure S6B,C). In this cohort, high expression of all NF-Y subunits is associated to a worse prognosis. In summary, prognostic implications are found with high levels of NF-YA in HCC and with all subunits when taking into account iCluster1 patients.

## 3. Discussion

HCC is the fourth chapter of our systematic evaluation of expression of NF-Y subunits in TCGA. As confirmed here, overexpression in epithelial tumors is becoming obvious, as previously reported by us and others. The abundance of NF-Y sites in the DEG of iCluster3 and in DEG common to all iClusters, as well as the increased expression of NF-YAs, are familiar features with respect to what we reported in other tumors. HCC-specific findings are: (i) there is overexpression of all subunits, but only NF-YA has a global clinical implication; (ii) there is no switch of NF-YA isoforms and no apparent role of NF-YAl; (iii) p53 mutations are associated with altered expression; and (iv) in iCluster1 patients, overexpression of all subunits is clinically relevant.

CCAAT boxes have been routinely found in promoters of genes overexpressed in cancer, including in RNA-seq data. CCAAT genes are clearly associated with pro-proliferative, cell-cycle and signaling GO terms [20,41,42]. Given the abundance of CCAAT in promoters in general [10] and the recent inclusion of NF-Y among the proteins dictating proper TSS selection [12], one might think that this is an “unspecific” phenomenon. It is clearly not so, and the case of HCC is illustrative. First, down-regulated genes are CCAAT-less, as is the case for the other down-regulated DEG we have analyzed so far in BRCA, LUSC, LUAD; second, because even among up-regulated genes, only iCluster3 tumors have enriched CCAAT genes. In this cohort, the parallel finding of E2F and GC boxes associated with cell-cycle and metabolic genes is the same for the other epithelial cancers we analyzed [41,42]. Interestingly, in a model of liver HepG2 tumorigenic stem cells Side Population (SP), NF-YA was shown to be overexpressed along with stem cell markers KLF4, SALL4, HMGA2, as well as cell-cycle genes [47]. Note, however, that the clinical data point to iCluster1 patients as those for whom NF-YA and NF-YC overexpression has an impact on the negative outcome of the disease. Overall, these data further strengthen the idea that highly specific CCAAT-centered promoter configurations exist in the genes activated by cancer. Rationalizing such differences in terms of TFBS promoter geometries—positioning and distancing—should be a priority.

The different isoforms of NF-YA and NF-YC share subunits-interaction and DNA-binding parts, leading to the formation of DNA-binding trimers with identical DNA-binding efficiencies. NF-YAs are dominant in normal livers and in HCC. This isoform is also dominant in the epithelial cancers analyzed so far. NF-YAl is expressed at marginal levels in normal livers and HCC, and it appears to be irrelevant to the development of hepatocellular carcinomas. This is in contrast with what we found in BRCA, in which a subset of Basal-like, Claudin-low tumors are NF-YAl^high^. This cohort corresponds to the most aggressive and metastasis-prone subset. NF-YAs lack 28/29 extra amino acids from exon 3; the NF-YC isoforms differ at the C-terminal due to alternative splicing of exons and donor/acceptor usage. As a result, isoforms of both subunits differ in the Gln-rich Trans-Activation Domains (TAD). The predominant NF-YC isoform is the 37 kD, which is highly expressed in HCC and in all other epithelial tissues (normal or tumors) that we have analyzed so far. An additional NF-YA isoform has been recently detected and studied as having an important role in Neuroblastomas, lacking additional portions of the TAD [48]. NF-YA isoforms are predicted, and to some extent also proven, to have differential activation potential [13,16]. We find here that HCC have the shortest versions of both NF-YA and NF-YC: our data is a step forward for the characterization of the configuration of this TF in HCC.

Unlike in breast and lung tumors, HFD subunits are robustly overexpressed in HCC at mRNA levels. Although neither is globally predictive of a different patient survival, association with the worst prognosis is detected in iClusters1 patients. This has not been found until now in the tumors we systematically analyzed. Their overexpression should be considered relative to the stoichiometry of the complex and localization of subunits. In fact, HFD subunits appear to be in excess with respect to NF-YA inside nuclei; in keeping with this, HFD levels are higher TPM-wise, as we found previously in BRCA, LUSC and LUAD. Limiting amounts of NF-YA would be consistent with its regulatory role in the formation of a trimeric, CCAAT-binding proficient complex. In addition, it was shown that NF-YB mediates the nuclear targeting of NF-YC [49,50]; in HCC, in the presence of higher levels of NF-YC in tumors, NF-YB levels also increase, possibly to guarantee higher HFD levels in the nuclei. In turn, they would be met by the higher levels of NF-YAs necessary for trimer formation. Related to this issue, removal of NF-YA, or NF-YB, by siRNA leads to an increase in the levels of the HFD subunits, or NF-YA, respectively [51]: this signals a negative feedback loop in the control of mRNA and protein levels. This would be globally interrupted in HCC, since all three subunits are upregulated.

Association of NF-Y subunits levels with tumors according to stages, inflammation and infection with viruses yielded modest differences: skewing was only visible in HBV tumors. A more robust link is detected with TP53 mutations. Since the vast majority (>85%) of lung tumors are mutated in p53 [52,53], a specific link with NF-Y expression levels could not emerge. This is visible in tumors with lower percentages of p53 mutations, such subtypes of BRCA (Basal-like/Triple Negative) and HCC. The fundamental difference is that most p53 mutations in BRCA tumors are missense located in the DNA-Binding Domain, with tumorigenic Gain-Of-Function (GOF) capacity. GOF TP53 are known to activate CCAAT-dependent target genes, and mutp53 and NF-Y interact directly [54,55,56,57,58,59]. In addition, the promoters of NF-Y subunit genes have CCAAT and GOF mutp53, typically present at high levels in tumors, and might be responsible for their activation. On the other hand, TP53 mutations in liver cancer are typically Loss-of-Function, with the exception of TP53 249, for which GOF was demonstrated [60]. The potential interplay between p53 status and NF-Y subunit overexpression is therefore clearly different in HCC, and other mechanisms must account for NF-Y subunit overexpression in these tumors. In this respect, HBV tumors have a higher rate of p53 mutations and shift gene expression patterns toward overexpression of early progenitors/fetal genes [61]. This is at least partially due to the activity of the HBV X transactivator, and in at least one case, the transactivation of HBV X appears to be NF-Y-dependent [35]; a possibility is that HBV X hijacks normal NF-Y function under conditions in which the normal cell-cycle blocking and proapoptotic functions of p53 are diminished or lost. TP53 mutations are enriched in iCluster3 tumors, which do show increased levels of the subunits; for this reason, we were somewhat surprised that association with a poor clinical outcome is not specifically found in iCluster3. Rather, overexpression of all subunits correlates with the worst prognosis only in iCluster1. Further work is required to find associations with other mutations specifically associated with iCluster1.

Finally, our results should be considered with respect to the treatment of HCC: a number of first- and second-line drugs are currently used to treat the disease [62,63]. In the case of Sorafenib, a tyrosine kinase inhibitor, efficacy is related to the mutational landscape of the tumor, being higher in TP53 wt tumors than in TP53 mut ones [64]. In this case, we expect that the same applies to the levels of NF-YAs, which correlate to the TP53 status. Due to the overall modest efficacy of these drugs in the long term management of patients, other drugs have been tested [65,66]. Thus, a possible outcome of our analysis is the evaluation of efficacies of established and novel drugs based on modifications of NF-YA levels, even tested in vitro, as well the development of anti-NF-Y compounds, as we have recently started to do, based on in silico screenings and in vitro testing [67].

## 4. Materials and Methods

### 4.1. TCGA HCC Data

As of May 2020, there are RNA-seq data on 373 Liver hepatocarcinomas [6] and 50 normal tissues in TCGA repository. We downloaded non-normalized tables from the http://firebrowse.org/ web page, and we obtained Transcript per Million (TPM), multiplying the scaled values by one million. From the firebrowse website, we also retrieved the merged clinical table containing the information about HCV-HBV and inflammatory states.

From https://xenabrowser.net/ [68] we retrieved the Illumina Human Methylation 450 table, somatic mutations (SNP and INDEL—MC3 public version) and Survival data, including Progression Free Interval (PFI) time records of the 364 patients currently available.

### 4.2. Classification of All TCGA HCC Tumors

The available TCGA classification of HCC in the three molecular iClusters comprises 183 tumors; we extended it to all HCC tumors by using the deep cancer subtype classification tool (DeepCC) version 0.1, which is based on the deep learning of functional spectra-quantifying activities [45]. We used the 183 samples already classified by TCGA as the training set, taking the scaled values of genes with standard deviation other than 0 as features in order to perform a supervised clustering of samples.

### 4.3. Global Gene Expression Analysis

Differentially Expressed Genes (DEG) analysis of RNA-seq data was performed using R package DESeq2, providing the raw counts as input [69]. The comparison of Tumor versus Normal expression fold change (FC) catalogued upregulation or downregulation, according to the FC value. LogFC and the corresponding false discovery rate (FDR) were measured by DESeq2. FDR < 0.01 and |log2FC| > 2 were set as inclusion criteria for DEG selection in tumor versus normal samples.

### 4.4. Gene Ontology, Pathway Enrichment and Transcription Factor Binding Site Analysis

We used KOBAS 3.0 (http://kobas.cbi.pku.edu.cn/anno_iden.php) for pathway enrichment analysis using the ENTREZ gene IDs. TFBS and de novo motif analysis were performed using the Pscan software tool [46].

### 4.5. Statistical Analysis

Graphs and statistical analysis were performed in the R studio environment (version 3.6.3) with DESeq2, ggplot2, ggsignif, reshape, ggpubr, clinfun, survival, suviminer, plyr, dplyr, datasets, data.table, heatmap.plus, and ReactomePA packages. P values were calculated with Wilcoxon rank sum test in R environment in order to compare the expression levels between two groups: samples normal and tumors, samples with minimal or no inflammation versus those with a detected inflammatory status, samples TP53 mutated versus TP53 wild type, and samples HCV-HBV negative versus HCV-HBV positive. Jonckheere’s Trend Test was used to verify significant gene expression trends in pre-ranked samples or grouped samples according to tumor stages, AFP serum levels and NF-Y expression. Survival analysis was performed according to the Kaplan-Meier analysis and log-rank test. Cox proportional hazard modelling of NFY subunit levels and covariates was calculated to determine their independent impact on patients’ survival and to estimate the corresponding hazard ratio [70].

## 5. Conclusions

All NF-Y subunits are overexpressed in HCC at the mRNA level, NF-YAs and NF-YC 37 kDa isoforms are associated with tumors with mutant TP53, and only NF-YAs globally correlate with the worst prognosis. In the up-regulated genes of the three clusters, only iCluster3 CCAAT promoters predominate, although the matrix is also present in commonly overexpressed genes. Overall, these data reinforce the idea that NF-YA is the regulatory subunit of the CCAAT-binding trimeric complex.

## Supplementary Materials

Supplementary materials can be found at https://www.mdpi.com/1422-0067/21/23/9157/s1. Supplementary Figure S1. Box plots showing NF-Y isoforms expression trend in samples ranked according to AFP serum levels. Supplementary Figure S2. HCC Tumor Inflammatory Signature -TIS. **A.** Boxsplots showing the 18 genes of the HCC Tumor Inflammatory Signature -TIS levels in tumors partitioned according to inflammatory status. **B**. Boxplots showing TIS signature genes in 5 groups of samples with none or minimal inflammation ranked according to NF-Y subunits expression levels. C. Same as B but samples with presence of inflammation are considered. Supplementary Figure S3. iClusters distribution of stage, age of onset and TP53 mutation. **A**. Number of samples belonging to each pathological stage in each iCluster. **B**. Median age at time of diagnosis based on iCluster classification. **C**. Pie plot showing the distribution of iClusters and TP53mut samples across HCC tumors. Supplementary Figure S4. NF-Y subunits expression in HCC iClusters. **A**. Box plots of expression levels of the three NF-Y subunits at gene level in the three iClusters and normal samples of the TCGA HCC dataset, measured in TPMs. **B**. Same as A, with the NF-YAs, NF-YAl, NF-YC 37 kDa, NF-YC 48 kDa and NF-YC 50 kDa isoforms. Supplementary Figure S5. TFBS enriched in promoters of down regulated genes. Pscan output of the top 30 TFBS enriched in promoters of down-regulated genes in each iCluster compared to normal samples. Supplementary Figure S6. Survival curve of NF-Y in HCC iClusters. Progression-Free-Interval curves of HCC tumors with stratification according to quartiles distribution of NF-Y subunits and splicing isoforms expression in (**A**) iCluster1 (**B**) iCluster2 (**C**) iCluster3. Supplementary Table S1. List of samples and HBV-HCV infection details. Supplementary Table S2. List of samples and stage, inflammation, AFP serum levels and iCluster classification details. Supplementary Table S3. Differential Expressed Genes.

## Abbreviations

TCGA	The Cancer Genome Atlas
NF-YAl	Nuclear Factor Y subunit A isoform long
NF-YAs	Nuclear Factor Y subunit A isoform short
NF-YB	Nuclear Factor Y subunit B
NF-YC	Nuclear factor Y subunit C
E2F	E2 Factor
KLF	Kruppel-Like Factor
TSS	Transcriptional Start Site
TF	Transcription Factor
TFBS	Transcription Factors Binding Sites
FDR	False Discovery Rate
EMT	Epithelial to Mesenchymal Transition
HFD	Histone Fold Domain
BRCA	Breast Carcinoma
LUSC	Lung Squamous Cells Carcinoma
LUAD	Lung AdenoCarcinoma
HCC	HepatoCellular Carcinoma
DEG	Differentially Expressed Genes
FDR	False Discovery Rate
FC	Fold Change
PFI	Progression Free Interval
