# Supplementary material for: NF-Y Overexpression in Liver Hepatocellular Carcinoma (HCC)
Source: Int J Mol Sci. 2020 Dec 1;21(23):9157. doi: 10.3390/ijms21239157 (PMC7731131; doi:10.3390/ijms21239157)
Supplement: Supplementary file 1 [file ijms-21-09157-s001.zip › SUPPLEMENTARY/Figures S1-6.pdf]

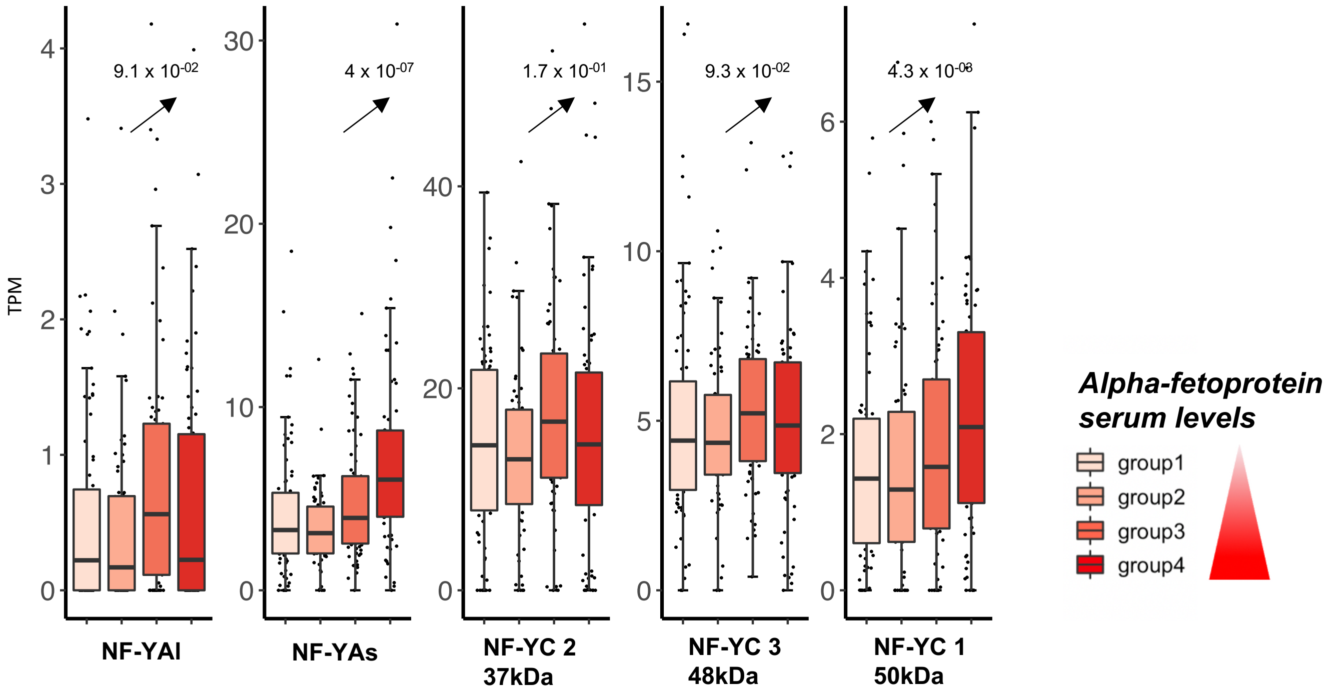
TPM
9.1 x 10-02
4 x 10-07
1.7 x 10-01
9.3 x 10-02
4.3 x 10-03
NF-YAl
NF-YAs
NF-YC 2
37kDa
NF-YC 3
48kDa
NF-YC 1
50kDa
Alpha-fetoprotein serum levels
group1
group2
group3
group4

Figure S1

A

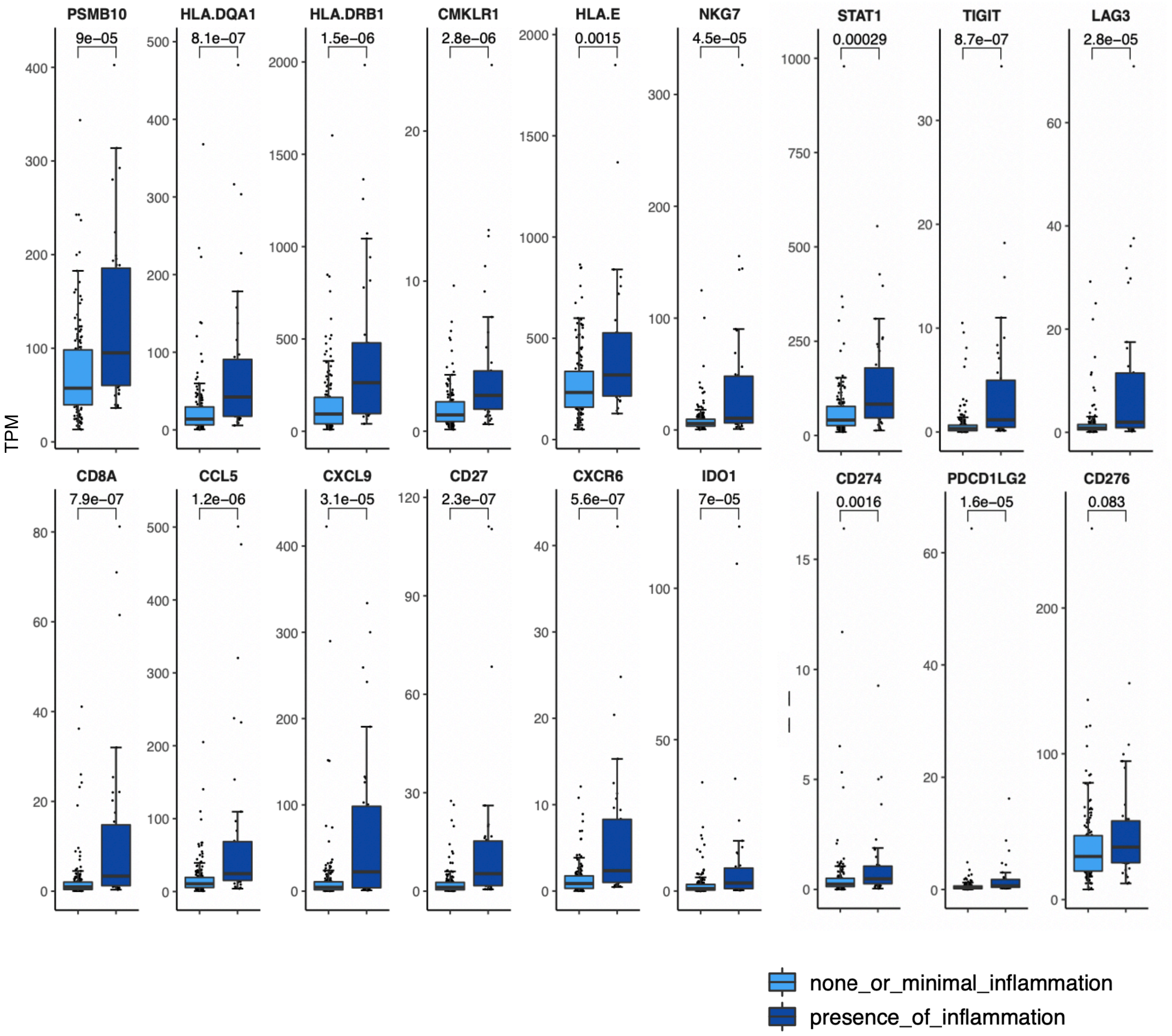
PSMB10
9e−05
HLA.DQA1
8.1e−07
HLA.DRB1
1.5e−06
CMKLR1
2.8e−06
HLA.E
0.0015
NKG7
4.5e−05
STAT1
0.00029
TIGIT
8.7e−07
LAG3
2.8e−05
TPM
CD8A
7.9e−07
CCL5
1.2e−06
CXCL9
3.1e−05
CD27
2.3e−07
CXCR6
5.6e−07
IDO1
7e−05
CD274
0.0016
PDCD1LG2
1.6e−05
CD276
0.083
none_or_minimal_inflammation
presence_of_inflammation

Figure S2

Samples with none or minimal inflammation N= 142

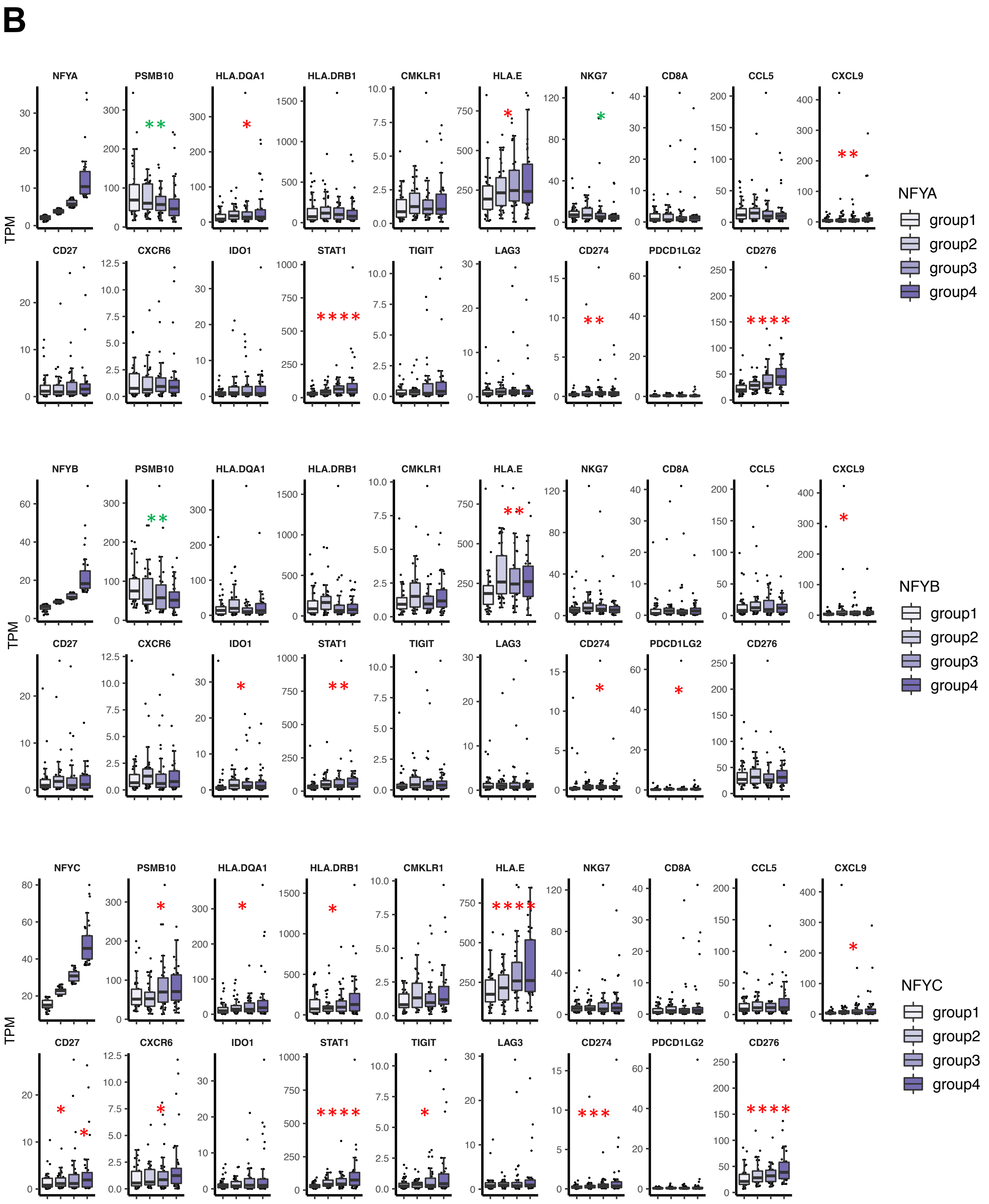

**** < 0.0001; *** < 0.001; ** < 0.01; * < 0.05
Green= JT test Hyp. Decreasing
Red= JT test Hyp. Increasing

Figure S2

Samples with inflammation N = 38

C

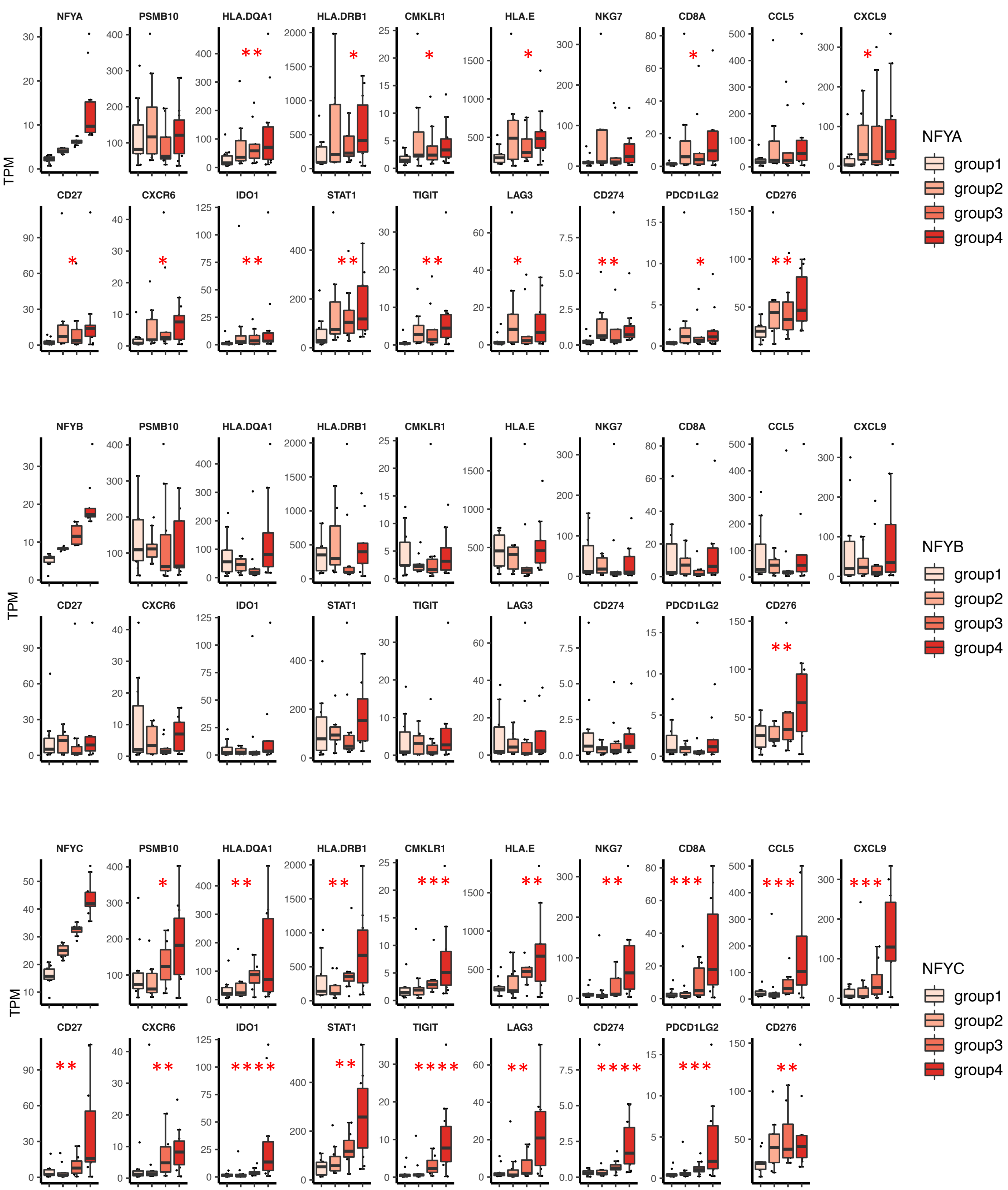

**** < 0.0001; *** < 0.001; ** < 0.01; * < 0.05
Red= JT test Hyp. Increasing

Figure S2

A

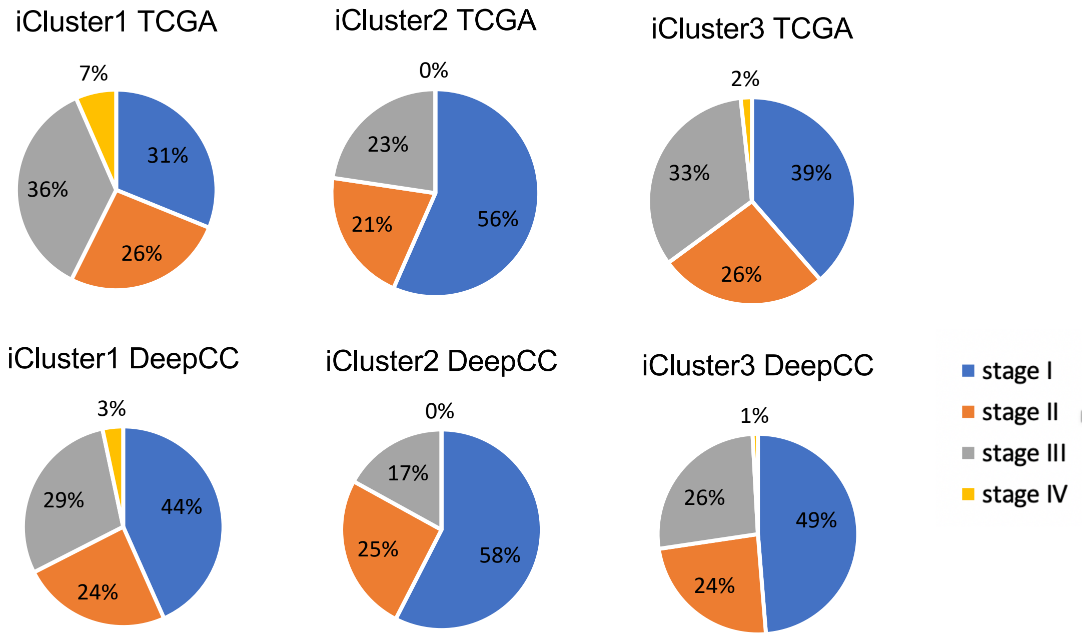

B

iCluster	Median age at diagnosis (TCGA)	Median age at diagnosis (DeepCC)
iCluster1	55	55
iCluster2	65	60
iCluster3	66	64

C

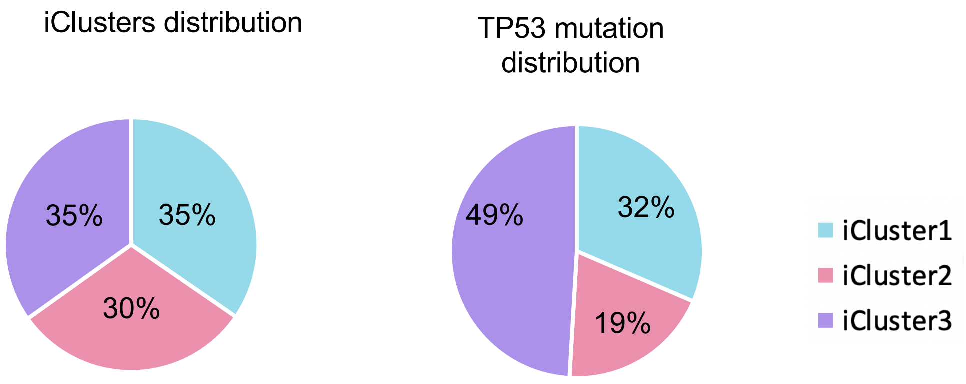

Figure S3

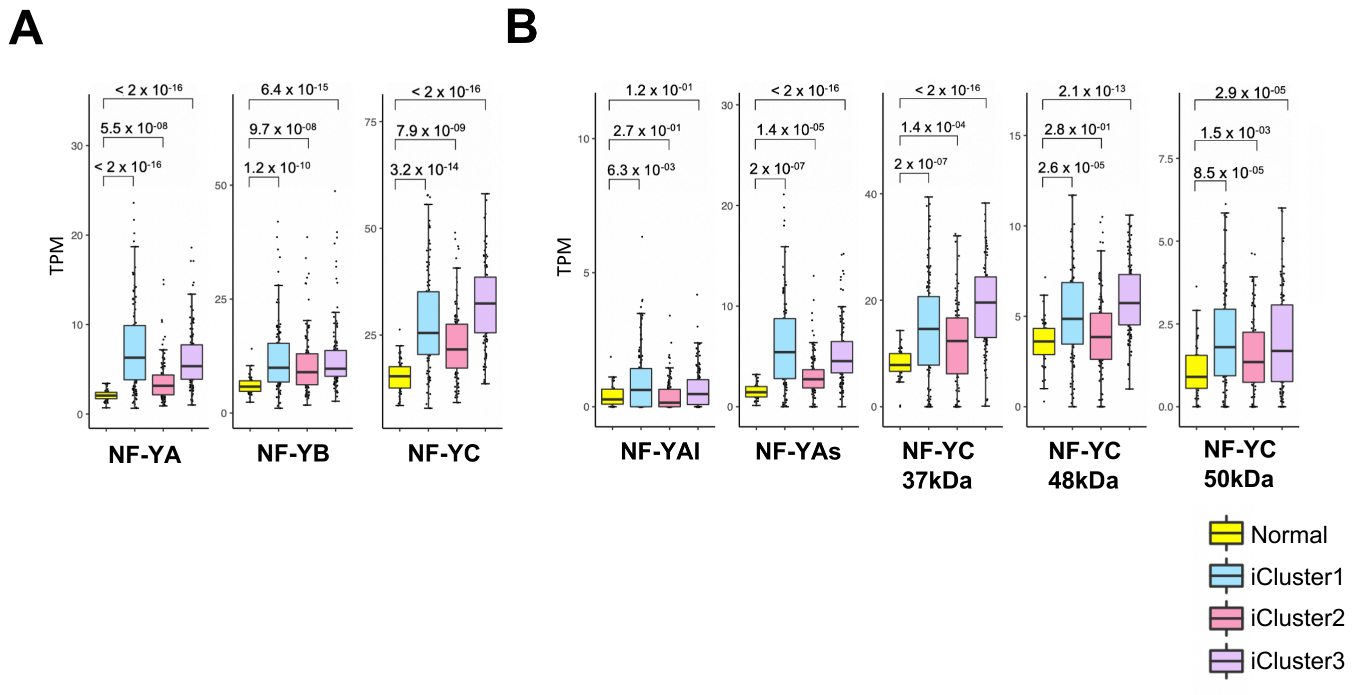

A
B
TPM
NF-YA
NF-YB
NF-YC
NF-YAl
NF-YAs
NF-YC 37kDa
NF-YC 48kDa
NF-YC 50kDa
Normal
iCluster1
iCluster2
iCluster3

Figure S4

iCluster1

635 TF profiles used

Matrix ID	Matrix Name	P-value
MA0484.1	HNF4G	7.79781e-15
MA0114.2	HNF4A	1.24569e-13
MA0115.1	NR1H2::RXRA	1.39488e-12
MA0114.1	HNF4A	3.93592e-11
MA0017.1	NR2F1	8.03237e-11
MA0114.3	Hnf4a	1.46372e-10
MA0042.2	FOXI1	2.59957e-10
MA0614.1	Foxj2	4.57476e-10
MA0031.1	FOXD1	4.95641e-10
MA0849.1	FOXO6	5.16972e-10
MA0848.1	FOXO4	6.09647e-10
MA0847.1	FOXD2	6.44667e-10
MA0033.2	FOXL1	8.29116e-10
MA0157.2	FOXO3	1.34971e-09
MA0036.2	GATA2	2.80156e-09
MA0148.1	FOXA1	4.58282e-09
MA0148.2	FOXA1	4.86247e-09
MA0047.1	Foxa2	5.04261e-09
MA0606.1	NFAT5	5.19548e-09
MA0593.1	FOXP2	5.69879e-09
MA0850.1	FOXP3	6.45439e-09
MA0512.1	Rxra	6.57393e-09
MA0108.2	TBP	7.5677e-09
MA0035.3	Gata1	8.57542e-09
MA0108.1	TBP	8.603e-09
MA0047.2	Foxa2	9.68668e-09
MA0613.1	FOXG1	2.80606e-08
MA0480.1	Foxo1	6.90124e-08
MA0851.1	Foxj3	7.83294e-08
MA0677.1	Nr2f6	9.48022e-08

iCluster2

635 TF profiles used

Matrix ID	Matrix Name	P-value
MA0108.1	TBP	0.000378551
MA0108.2	TBP	0.000385172
MA0503.1	Nkx2-5(var.2)	0.000667105
MA0116.1	Znf423	0.000703257
MA0036.2	GATA2	0.000994206
MA0035.2	Gata1	0.00125414
MA0105.3	NFKB1	0.00214568
MA0482.1	Gata4	0.00252953
MA0035.3	Gata1	0.00339005
MA0092.1	Hand1::Tcf3	0.0039059
MA0083.1	SRF	0.0042211
MA0140.1	Tal1::Gata1	0.00439039
MA0154.2	EBF1	0.00493726
MA0083.2	SRF	0.00530993
MA0003.2	TFAP2A	0.00561995
MA0528.1	ZNF263	0.00645778
MA0105.2	NFKB1	0.00756551
MA0083.3	SRF	0.00772832
MA0149.1	EWSR1-FLI1	0.00786241
MA0524.2	TFAP2C	0.00873574
MA0524.1	TFAP2C	0.00924599
MA0766.1	GATA5	0.0109919
MA0106.1	TP53	0.0119032
MA0112.2	ESR1	0.0119288
MA0056.1	MZF1	0.0126301
MA0140.2	GATA1::TAL1	0.0135694
MA0811.1	TFAP2B	0.0137575
MA0154.1	EBF1	0.0141916
MA0090.1	TEAD1	0.015917
MA0037.2	GATA3	0.0169673

iCluster3

635 TF profiles used

Matrix ID	Matrix Name	P-value
MA0108.2	TBP	8.24939e-07
MA0108.1	TBP	9.65902e-07
MA0528.1	ZNF263	1.00774e-05
MA0149.1	EWSR1-FLI1	6.86583e-05
MA0080.3	Spi1	0.000114279
MA0140.1	Tal1::Gata1	0.000160893
MA0508.1	PRDM1	0.000260808
MA0606.1	NFAT5	0.000452471
MA0503.1	Nkx2-5(var.2)	0.000971339
MA0080.4	SPI1	0.00101472
MA0152.1	NFATC2	0.00144004
MA0154.2	EBF1	0.00206836
MA0090.1	TEAD1	0.00207818
MA0119.1	NFIC::TLX1	0.00214173
MA0140.2	GATA1::TAL1	0.00276256
MA0107.1	RELA	0.00284593
MA0498.1	Meis1	0.00300246
MA0105.3	NFKB1	0.00335099
MA0461.1	Atoh1	0.0039535
MA0625.1	NFATC3	0.00442684
MA0090.2	TEAD1	0.00483781
MA0036.2	GATA2	0.00542235
MA0154.1	EBF1	0.00619301
MA0513.1	SMAD2::SMAD3::SMAD4	0.0065238
MA0161.1	NFIC	0.00734737
MA0035.2	Gata1	0.00761295
MA0035.3	Gata1	0.00786626
MA0624.1	NFATC1	0.0101679
MA0081.1	SPIB	0.0109832
MA0471.1	E2F6	0.0113402

Figure S5

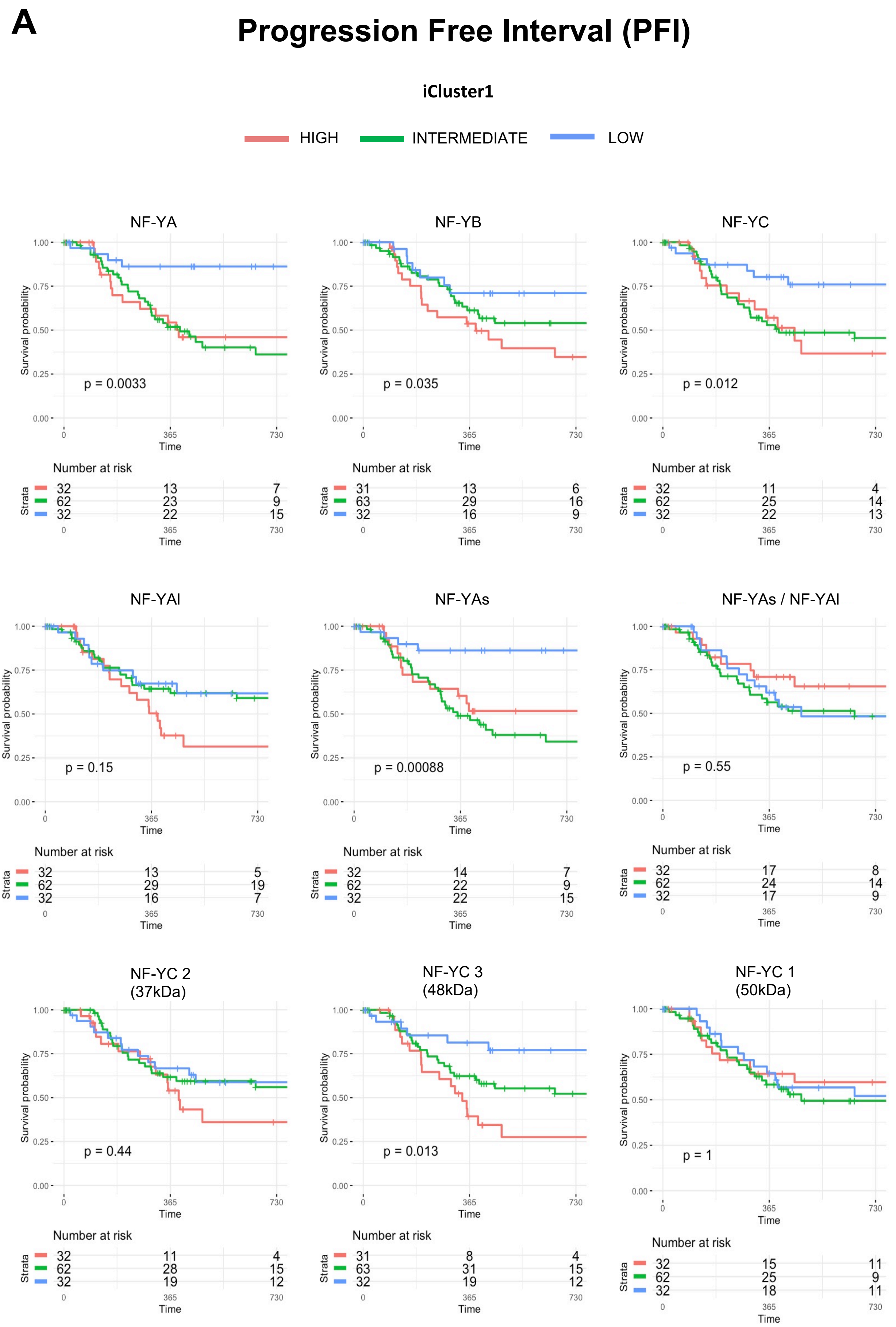
A
Progression Free Interval (PFI)
iCluster1
HIGH
INTERMEDIATE
LOW
NF-YA
p = 0.0033
NF-YB
p = 0.035
NF-YC
p = 0.012
NF-YAl
p = 0.15
NF-YAs
p = 0.00088
NF-YAs / NF-YAl
p = 0.55
NF-YC 2 (37kDa)
p = 0.44
NF-YC 3 (48kDa)
p = 0.013
NF-YC 1 (50kDa)
p = 1
Survival probability
Time
Number at risk
Strata

Figure S6

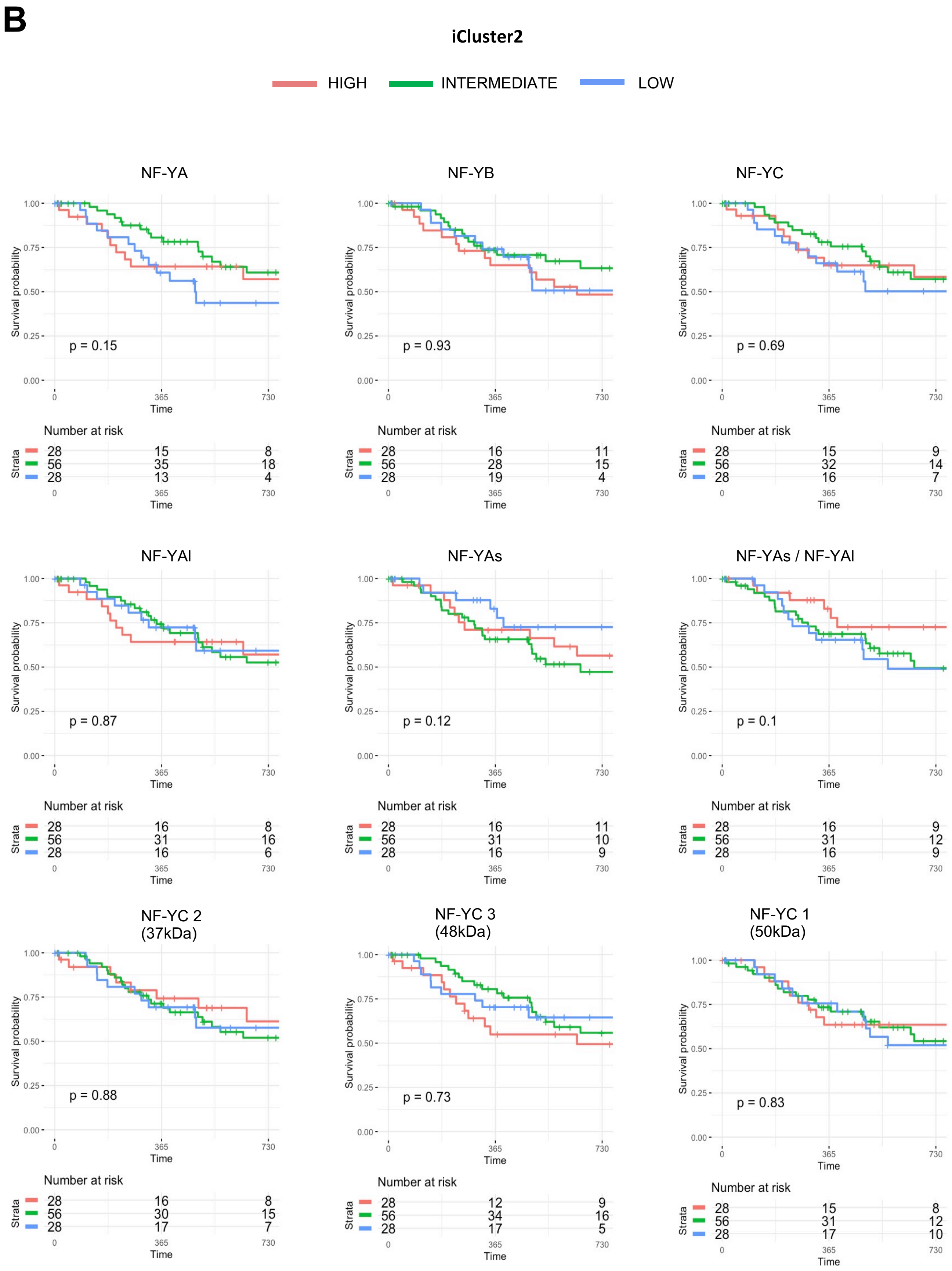
B
iCluster2
HIGH
INTERMEDIATE
LOW
NF-YA
p = 0.15
Number at risk
28 15 8
56 35 18
28 13 4
NF-YB
p = 0.93
Number at risk
28 16 11
56 28 15
28 19 4
NF-YC
p = 0.69
Number at risk
28 15 9
56 32 14
28 16 7
NF-YAl
p = 0.87
Number at risk
28 16 8
56 31 16
28 16 6
NF-YAs
p = 0.12
Number at risk
28 16 11
56 31 10
28 16 9
NF-YAs / NF-YAl
p = 0.1
Number at risk
28 16 9
56 31 12
28 16 9
NF-YC 2
(37kDa)
p = 0.88
Number at risk
28 16 8
56 30 15
28 17 7
NF-YC 3
(48kDa)
p = 0.73
Number at risk
28 12 9
56 34 16
28 17 5
NF-YC 1
(50kDa)
p = 0.83
Number at risk
28 15 8
56 31 12
28 17 10
Survival probability
Strata
Time
0
365
730

Figure S6

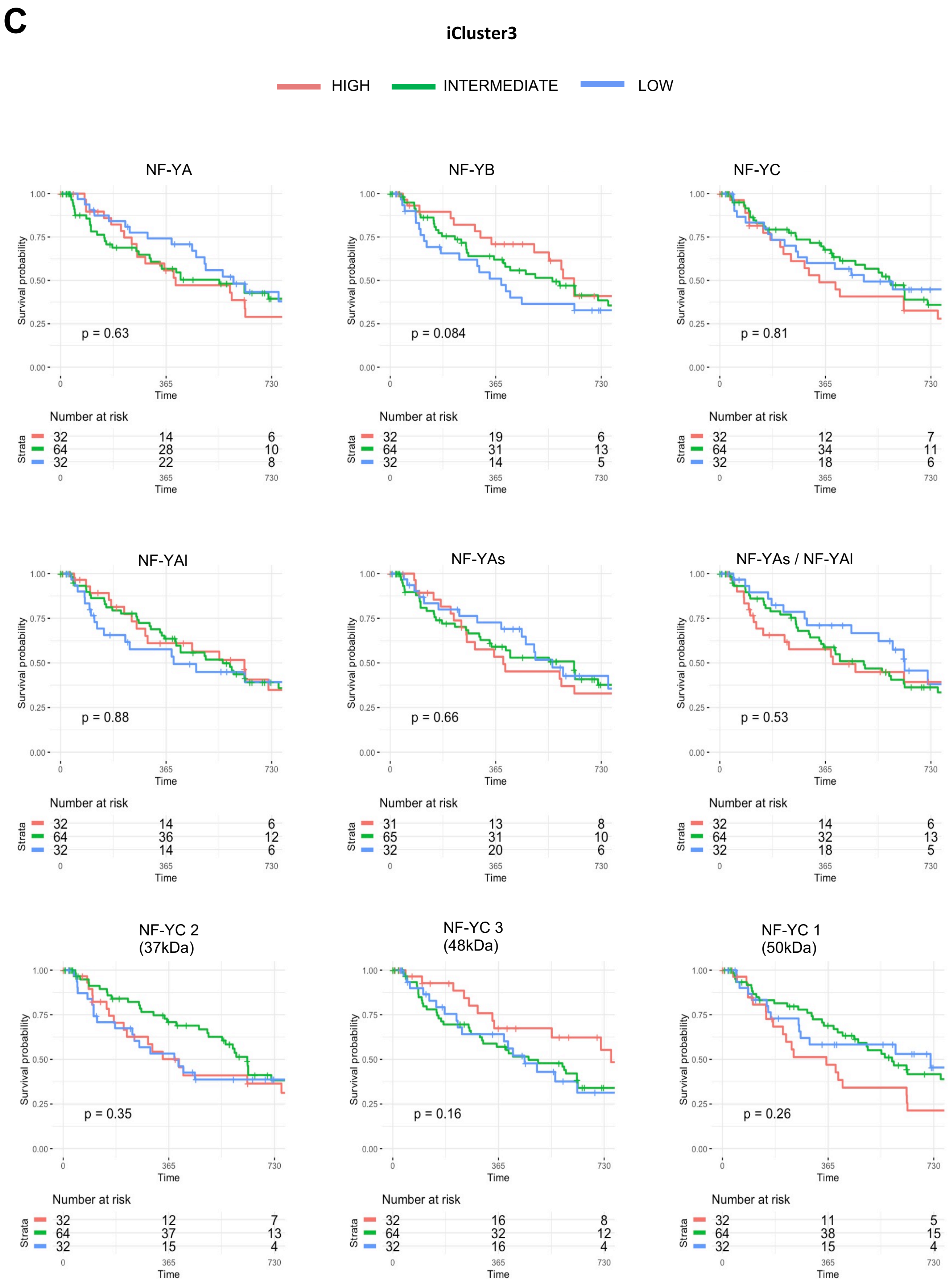
C
iCluster3
HIGH
INTERMEDIATE
LOW
NF-YA
p = 0.63
Number at risk
32 14 6
64 28 10
32 22 8
NF-YB
p = 0.084
Number at risk
32 19 6
64 31 13
32 14 5
NF-YC
p = 0.81
Number at risk
32 12 7
64 34 11
32 18 6
NF-YAl
p = 0.88
Number at risk
32 14 6
64 36 12
32 14 6
NF-YAs
p = 0.66
Number at risk
31 13 8
65 31 10
32 20 6
NF-YAs / NF-YAl
p = 0.53
Number at risk
32 14 6
64 32 13
32 18 5
NF-YC 2 (37kDa)
p = 0.35
Number at risk
32 12 7
64 37 13
32 15 4
NF-YC 3 (48kDa)
p = 0.16
Number at risk
32 16 8
64 32 12
32 16 4
NF-YC 1 (50kDa)
p = 0.26
Number at risk
32 11 5
64 38 15
32 15 4
Survival probability
Time
Strata

Figure S6
